# Pride and guilt as place-based affective antecedents to pro-environmental behavior

**DOI:** 10.3389/fpsyg.2022.1084741

**Published:** 2023-01-19

**Authors:** Nathan J. Shipley, Carena J. van Riper, William Stewart, Maria Chu, Richard C. Stedman, Florin Dolcos

**Affiliations:** ^1^Department of Natural Resources and Environmental Sciences, University of Illinois at Urbana-Champaign, Urbana, IL, United States; ^2^Department of Recreation, Sport and Tourism, University of Illinois at Urbana-Champaign, Champaign, IL, United States; ^3^Agricultural and Biological Engineering, University of Illinois at Urbana-Champaign, Urbana, IL, United States; ^4^Department of Natural Resources and the Environment, Cornell University, Ithica, NY, United States; ^5^Department of Psychology, University of Illinois at Urbana-Champaign, Beckman Institute for Advanced Science and Technology, Champaign, IL, United States

**Keywords:** place attachment, place meaning, emotion, pro-environmental behavior, environment - agriculture, structural equation modeling

## Abstract

The interrelated concepts of place attachment and place meaning are antecedents to pro-environmental behavior and essential for supporting decisions that foster relationships between people and places. Previous research has argued that affect is instrumental in conceptualizing place-related phenomena but has not yet been considered in terms of discrete emotions. We disentangled the empirical relationships between concepts of place and the emotions of pride and guilt to understand how they collectively contributed to individuals’ decisions about environmental sustainability. Specifically, we conducted an online survey of residents living in the Midwestern US and asked questions about their attachments to places and their place-related behavior. We then tested a latent variable path model with first- and second-order factors that shaped the behavioral intentions of survey respondents, as well as evaluated the psychometric properties of a place meaning scale, to uncover the range of reasons why human-nature relationships were formed. Our findings show that multiple place meanings predicted place attachment, which in turn predicted the discrete emotions of pride and guilt. Place attachment, pride, and guilt positively correlated with pro-environmental behavior. We also observed that the relationships between multi-dimensional conceptualizations of place attachment and behavioral intentions were partially mediated by pride but not guilt, as hypothesized in response to the broaden and build theory of positive emotions. This study develops theoretical insights to clarify how cognitive-emotional bonding can lead people to behave in more environmentally friendly ways.

## 1. Introduction

The scholarship surrounding concepts of place is an evolving research area in environmental psychology (Trentelman, 2009; Lewicka, 2011; Raymond et al., 2021) that fits a variety of paradigmatic approaches (Stedman and Beckley, 2007; Williams and Patterson, 2007). Across this body of literature, one focus has been directed toward place attachment, defined as the evaluative strength of emotional bonds to a place (Low and Altman, 1992; Williams and Vaske, 2003). Another thread of this literature has focused on place meanings, defined as the symbolic and representative elements of a place (Gustafson, 2001; Manzo, 2005; Stedman, 2008; van Riper et al., 2016). Scholars have sought to integrate place meanings and attachment to advance knowledge of social-ecological systems (Stedman, 2003; Oteros-Rozas et al., 2015; Masterson et al., 2017) and pro-environmental behavior (PEB) (Scannell and Gifford, 2010; Devine-Wright, 2013; Larson et al., 2018), defined as actions that are intended to benefit the environment (Steg and Vlek, 2009). We seek to build on this previous work by quantitatively examining the integration of meanings and attachment, as well as their role in explaining pro-environmental behavior.

Concepts of emotion are integral to understanding both place attachment and place meaning but have previously been implicit in the conceptualization and measurement of human-place bonding research. Place attachment, in particular, has been treated as a construct that is comprised of affect, in addition to other dimensions such as place dependence, place identity, and social bonding (Jorgensen and Stedman, 2001; Kyle et al., 2004; Ramkissoon et al., 2013; Zhang et al., 2014; van Riper et al., 2019). By contrast, contemporary emotion research has examined affective phenomena in nuanced ways by distinguishing among *emotional states* described as the physiological and neurological processes that arise in response to stimuli, *feelings* that refer to the subjective and conscious experience that accompanies an emotional state, and *emotional concepts* that encompass semantic knowledge of emotional states, which includes human abilities to think, make attributions, and verbally communicate (Barrett et al., 2007). To bridge these parallel areas of inquiry, deeper knowledge of how affective phenomena function should be established in the place attachment literature. The discrete emotions of pride and guilt warrant particular research attention, as reflected by their pivotal roles in the moral norm activation model (Schwartz, 1977) and value-belief-norm theory of environmentalism (Stern et al., 1999), which posit that morality - encompassing both pride and guilt - is a direct antecedent to environmental stewardship behaviors. That is, both pride and guilt provide a starting point for understanding how emotions can be more effectively measured and theoretically positioned as powerful triggers for behavioral decisions (Zhang et al., 2014; Shipley and van Riper, 2022).

## 2. Literature review

### 2.1. Conceptualizing the relationship between place meanings and place attachment

Previous research has suggested that place attachment builds over time (e.g., Hay, 1998) and arises from the meanings that are imbued in a place (Stedman, 2003). This process of strengthening connections between people and places has been investigated in a variety of natural resource management contexts. For example, Stedman (2006) found that place meanings associated with a sense of community and environmental quality predicted respondents’ levels of attachment to their homes in northern Wisconsin, USA. Similarly, Larson et al. (2018) demonstrated that, amongst outdoor recreationists, place meanings were associated with both the environmental and sociocultural domains of place attachment. These studies provide empirical evidence for a hypothesizing a correlational relationship between dimensions of place meanings (e.g., environmental, sociocultural) and place attachment. Other scholars have used mixed methods to quantify place meanings that are regionally representative (Wynveen et al., 2012). For instance, Evans et al. (2022) conducted in-depth interviews and focus groups with residents in Illinois and Iowa, USA and then developed a psychometric scale including eight place meanings that reflected the reasons why residents were connected to places at a regional scale. These authors highlighted the importance of qualities such as outdoor living, agricultural pride, and a small town feeling in characterizing landscapes of the Midwestern USA. This body of past work has indicated that place meanings necessarily vary across contexts but can be quantified and generalized to broader scales in ways that reveal the intricacies of how people connect to their environments.

### 2.2. Connecting pro-environmental behavior with concepts of place

The study of pro-environmental behavior (PEB) has been generally inspired by cognitive psychological frameworks that depict humans as rational, goal seeking, and information-based actors (Ajzen, 1991, 2020). Yet recent work in affective psychology has opened pathways that hold promise to explain a wider array of human behaviors including those that may appear counter-intuitive, frivolous, or altruistic (Stern, 2000). With place research having a history of inquiry that integrates emotions, feelings, and cognition (Lewicka, 2011), this research poses questions about how best to integrate place, emotions and PEB. In addition, place has a normative quality, that has been characterized by Cresswell (2004) as recognition of some features as being “in place” and others as being “out of place.” In this sense, place is aspirational, in tha, people work to make their environments into places that “should be”. For example, Morse et al. (2014) were explicit about the communication potential of place to influence ways in which nearby landowners care for, and steward, their own land. Building on this study, we were inspired by possibilities to make space for understanding how emotions influence PEB, as well as testing the degree to which the aspirational qualities of place attachment and meanings also lead to environmentally-friendly outcomes.

Pro-environmental behavior (PEB) can be conceptualized a multi-dimensional construct. The extant literature has evaluated PEBs such as recycling, reducing water and electricity consumption, supporting climate change policy, and participating in or donating to environmental groups (Kollmuss and Agyeman, 2002; van Riper and Kyle, 2014; Lange and Dewitte, 2019; Daryanto and Song, 2021). This range of activities has been distilled into more cohesive themes (Stern, 2000; Zhou et al., 2020). For example, Stern et al. (1999) identified four dimensions of PEB that included environmental activism, environmental citizenship, policy support, and private-sphere behaviors. Building on this work, Larson et al. (2015) engaged rural landowners in discussions to understand how they enhanced the quality of local environments in New York, USA. These authors showed that PEB spanned the dimensions of conservation lifestyles, environmental citizenship, social environmentalism, and land stewardship behaviors, while also indicating that careful attention should be paid to the structure of PEB as a first- or second-order factor model. Given the importance of socialization in both PEB and human-place bonding (Kyle and Chick, 2007), social environmentalism has also been identified as an important aspect of place attachment, alongside actions that are relevant to public and private domains (Landon et al., 2018; van Riper et al., 2019; Winkler-Schor et al., 2020; Golebie et al., 2021).

There is long-standing evidence that higher levels of place attachment are associated with greater engagement in PEB (Vaske and Kobrin, 2001; Lewicka, 2011; Ramkissoon et al., 2013; Manning et al., 2022). In the context of a Canadian national park, place attachment of visitors was positively correlated with reported PEBs (Halpenny, 2010; Scannell and Gifford, 2010). Similar patterns emerged among nature-based recreationists and property owners from rural counties in NY, in that as their place attachment increased, so too did PEB in small but significant ways (Larson et al., 2018). Other scholars have observed mixed and at times non-significant associations between place attachment and behavior. For example, Scannell and Gifford (2010) sought to clarify how two dimensions of place attachment were related to PEB amongst residents of two towns in British Columbia, Canada that differed in socio-economic status and environmental condition. These authors found that the strength of connections to natural features predicted PEB, and reinforced previous research suggesting the role of the physical environment (Stedman, 2003; Stedman and Ingalls, 2014) and its broader social milieu (Stokowski, 2002) were instrumental in understanding human-place bonding.

### 2.3. Anticipated pride and guilt in relation to concepts of place

Emotions are instrumental in conceptualizing place-related phenomena, yet discrete emotions have been understudied in previous place attachment research. Pride and guilt are both self-conscious, discrete emotions that arise from prescriptive self-evaluations of an individual’s behavior (Tangney et al., 2007). Pride is a positive feeling that is experienced when behavior aligns with moral values, which promotes engagement in pro-social behaviors as well as encourages the repetition of such behavior (Tracy and Robins, 2007). Guilt, on the other hand, is a negative feeling experienced when behavior does not align with one’s moral inclinations, which motivates behaviors focused on repairing and discontinuing harm (Tangney and Dearing, 2003). Pride and guilt can arise as anticipated feelings that are expected to be felt in the future and informed by past and/or present experiences (Kahneman, 2000; Loewenstein and Lerner, 2003). When an individual anticipates feeling proud or guilty about engaging in PEB that influences places, it is possible that these anticipated feelings can be associated with existing attachments (Lewicka, 2011). Therefore, place attachment may help to establish part of the foundational past experiences that include feelings of pride and guilt.

A growing body of work has demonstrated that pride and guilt are instrumental in explaining behavioral patterns (Bissing-Olson et al., 2016; Schneider et al., 2017; Adams et al., 2020; Shipley and van Riper, 2022), as evidenced by the norm activation model that posits moral concerns are direct antecedents to PEB (Schwartz, 1977). Within this body of work there is mixed evidence concerning whether pride or guilt has a stronger effect on PEB (Onwezen et al., 2013; Han et al., 2017; Adams et al., 2020); however, more recent research suggests that anticipated pride may have a stronger effect on PEB than anticipated guilt (Shipley and van Riper, 2022). This scholarship has drawn upon the broaden and build theory of positive emotions (Fredrickson, 2001) as a framework to elucidate the differing effects that pride and guilt may have on PEB (Bissing-Olson et al., 2016). This theory posits that negative emotions such as guilt influence behavior by narrowing tendencies to a specific set of behavioral responses (Dahl et al., 2005). For example, previous research has indicated that environmental programs where people “opt-out” rather than “opt-in” are more sustainable because of increased feelings of guilt (Theotokis and Manganari, 2015). In contrast, positive emotions such as pride influence behavior by broadening awareness, which in turn, expands the range of actions a person might pursue. For example, researchers have found that feelings of pride were associated with increased levels of public self-awareness linked to more pro-social behaviors (Hwang and Lee, 2019). Because feelings of pride encourage engagement in new behaviors and guilt is associated with reductions in harmful behaviors, it is likely that pride and guilt have different roles as mediators of the relationship between place attachment and PEB.

## 3. Hypothesis development

**Figure 1:**
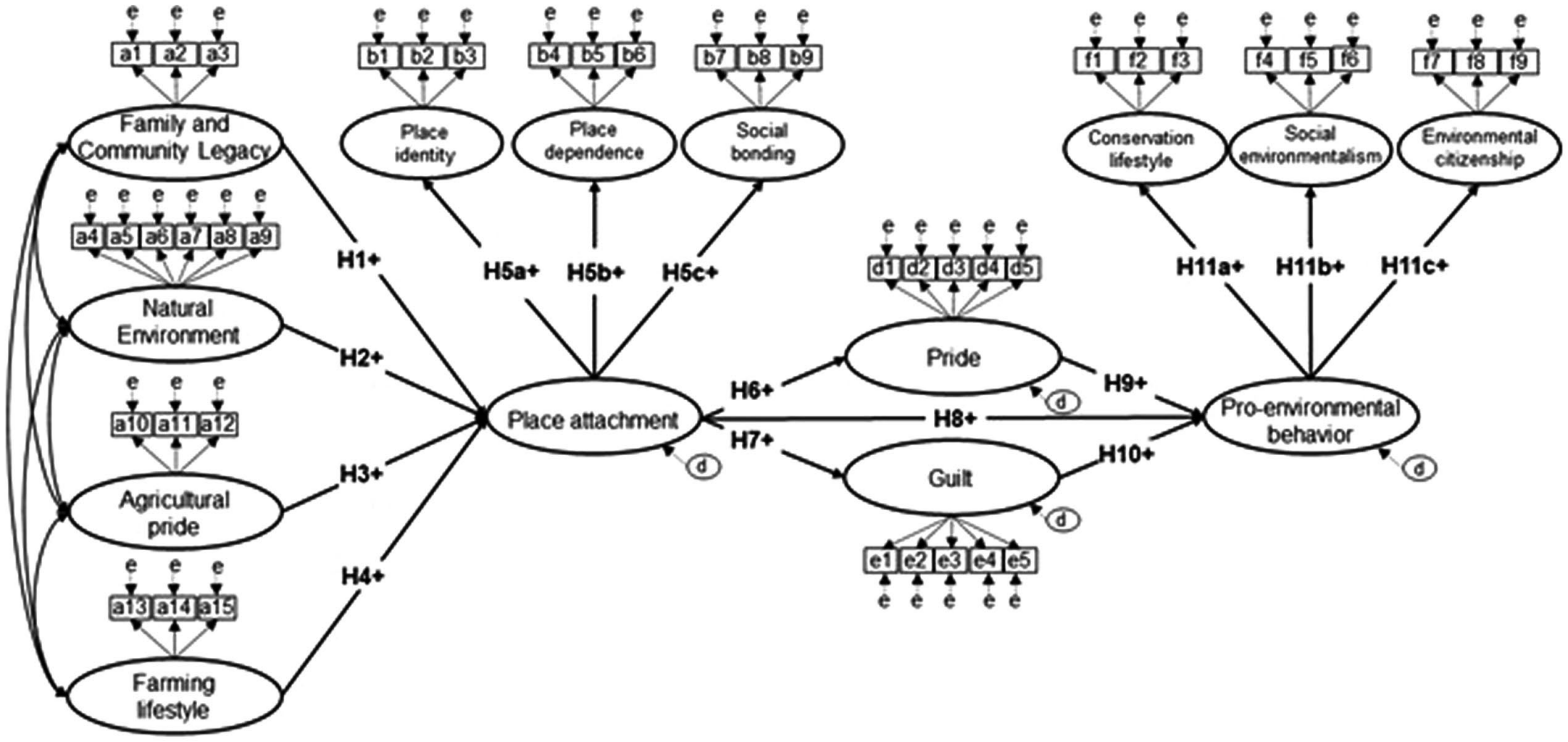
Hypothesized relationships among place meanings, place attachment, anticipated emotions, and pro-environmental behavior.

The purpose of this study was to test the linkages among place meanings, place attachment, anticipated emotions, and PEB. Drawing on previous research, we tested 11 hypotheses (see Table 1) using a latent variable structural equation model (see Figure 1). Given that previous research has suggested place attachment is formed through a process of imbuing place with meanings (Stedman, 2003; Rajala et al., 2020), we hypothesized that four dimensions of place meanings that characterized a rural area would positively predict place attachment (H1–H4). Place attachment is increasingly conceptualized as a multi-dimensional construct so we hypothesized that place attachment would be reflected by three dimensions of place identity, place dependence, and social bonding (H5a-H5c). Anticipated emotions are forecasted feelings shaped by previous experiences (Loewenstein and Lerner, 2003; Baumeister et al., 2007) so we hypothesized that place attachment would positively predict pride (H6) and guilt (H7). Notably, to the authors knowledge, no studies to date have empirically evaluated the effects of pride and guilt on place attachment and pride and guilt. Previous studies have suggested that higher levels of place attachment are associated with higher levels of engagement in PEB (Halpenny, 2010; Scannell and Gifford, 2010). Therefore, we hypothesized that place attachment would positively predict PEB (H12). We also hypothesized that PEB would be positively predicted by the anticipated emotions of pride (H9) and guilt (H10) in response to empirical evidence generated by Shipley and van Riper (2022). Lastly, given previous research on multiple dimensions of PEB (Larson et al., 2018), we hypothesized this construct would be comprised of conservation lifestyles (H11a), social environmentalism (H11b), and environmental citizenship (H11c). As illustrated in our hypothesized model, place attachment and PEB are multidimensional constructs that include second-order factors. We also tested the indirect effects of place attachment on PEB through anticipated pride and guilt. Specifically, we expected that place attachment would have a positive indirect effect on PEB through anticipated pride but not through anticipated guilt.

**Table 1:** Previous research supporting hypothesized relationships among place meanings, place attachment, anticipated emotions, and pro-environmental behavior.

Hypotheses	Rationale	Supporting literature
H1–H4	The formative process of place attachment is imbued by the meanings of places. People can be attached to the same place for multiple reasons (i.e., meanings) so the four meanings measured in this study will positively predict place attachment.	Stedman, 2003; Kyle et al., 2004; Larson et al., 2018; Rajala et al., 2020
H5a,b,c	A second order latent factor of place attachment includes the three dimensions of place identity, place dependence, and social bonding.	Kyle et al., 2005; Ramkissoon et al., 2013
H6–H7	Anticipated emotions are forecasted feelings that are shaped by previous experiences and stronger place attachment results in heightened anticipated emotions about future behavior. Therefore, place attachment positively predicts anticipated feelings of pride and guilt.	Kahneman, 2000; Loewenstein and Lerner, 2003; Baumeister et al., 2007
H8	Higher levels of place attachment have direct and positive effects on pro-environmental behavioral intentions.	Halpenny, 2010; Scannell and Gifford, 2010
H9–H10	Anticipated emotions are a response to intended actions, in that having higher levels of anticipated pride and guilt positively predicts higher levels of intentions to engage in pro-environmental behaviors.	Onwezen et al., 2013; Han et al., 2017; Wang and Wu, 2016; Schneider et al., 2017
H11a,b,c	A second order latent factor of pro-environmental behavior includes three dimensions, including conservation lifestyle, environmental citizenship, and social environmentalism.	Larson et al., 2018

## 4. Materials and methods

### 4.1. Study context and data collection

Most research on concepts of place and PEB have been conducted in either high-amenity or home environments. In contrast, the context for this study was a working agricultural watershed directed at understanding the presence and possibilities for PEB within a type of environment that accounts for a significant portion of global land use. Our data were collected from residents living within or near the Kaskaskia River Watershed. This region spans parts of central and southern Illinois (USA), constituting roughly 10 percent of the state. Over 70 percent of land area in the watershed is devoted to agriculture, consisting largely of cultivated corn and soybean crop (Homer et al., 2015). The region also contains forested areas (16 percent), urban land cover (9 percent), and wetlands (2 percent; Metzke and Hinz, 2017). The primary bodies of water include the Kaskaskia River and two large U.S. Army Corps of Engineers reservoirs. Much of the watershed is defined as rural yet larger urban centers such as Saint Louis MO, as well as Decatur and Champaign-Urbana in IL are either within the watershed boundary or in an adjacent county. Understanding how a geographically diverse population values the region and responds to threats is important, not only because the watershed provides diverse benefits, such as crop production, flood control, and recreation, but also because it faces numerous threats such as erosion, siltation and sedimentation that benefit people from both contexts (Shipley et al., 2020). Additionally, the rural way of life found in context such as Illinois is increasingly threatened by landscape change such as land conversion, tourism, and agricultural intensification (Strauser et al., 2019; Foelske and van Riper, 2020) that warrant research attention.

We collected data from an aggregated online panel of respondents living in the Kaskaskia River Watershed that was assembled by Qualtrics. Our decision to use an online panel was driven by concerns about declining response rates in traditional mail-back surveys (Stedman et al., 2019), particularly in rural contexts (Coon et al., 2020), and paralleled rises in the use of panel data to study psychological phenomena related to natural resource management issues (e.g., Larson et al., 2019; van der Linden et al., 2019; Goldberg et al., 2020; Landon et al., 2020). Our panel was comprised of residents living in the area defined by 22 counties in Southern Illinois that were completely or partially within the boundary of the Kaskaskia River Watershed. To maximize the generalizability of our sample, we implemented demographic quotas to ensure that the gender, age, and race of respondents aligned with Illinois residents in the U.S. Census (U.S. Census Bureau, 2012). A total of 786 residents were invited to participate in this study in 2020. The final sample size was 617 after dropping 128 duplicate responses and 41 respondents who failed an attention-check survey item. This study was reviewed and approved by the University of Illinois Institutional Review Board.

### 4.2. Survey measures

All survey items and response categories are displayed in Table 2. We measured place attachment across three dimensions that included nine survey items adapted from previous research (Kyle et al., 2004). We tailored our scale to focus on the area where respondents lived. We measured place meanings using 15 items that spanned four dimensions. These items were modified from a previous study focused on measuring place meanings of residents who live in rural communities undergoing landscape change (Evans et al., 2022). Our adaptations to the scale were informed by findings gathered from previously conducted interviews that sampled farmers living in the Kaskaskia River watershed and focused on their place meanings. This phase of our research enabled us to understand the narratives and priorities of residents who live in rural communities that are experiencing growth due to urban encroachment (Leitschuh et al., 2022; Shipley et al., 2022). Anticipated pride and guilt were measured by 10 survey items drawn from previous scales (Onwezen et al., 2013; Shepherd et al., 2013; Wang and Wu, 2016; Han et al., 2017). Pro-environmental behavior was measured by nine survey items that reflected three dimensions highlighted in previous research (Larson et al., 2015; Landon et al., 2018).

**Table 2:** Means (x¯), standard deviations (SD), internal consistencies, z-values, and standardized factor loading scores for scale items measuring place attachment, place meanings, anticipated emotions, and pro-environmental behaviors among respondents.

	x¯ *(SD)*	λ	*z*-value
**Place meanings** ^**2**^			
*Family and community legacy* (*α* = 0.88, CR = 0.88, AVE = 0.66)	*3.73 (0.87)*		
A1. Local community where residents know each other	3.92 (0.97)	0.79	22.74
A2. Shared community history and culture	3.70 (0.97)	0.85	25.44
A3. Close personal relationships in the community	3.72 (1.04)	0.83	24.60
A4. Opportunities to create a legacy that supports future generations in my family	3.58 (1.04)	0.77	21.85
*Natural environment* (*α* = 088, CR = 0.85, AVE = 0.58)	*3.79 (0.84)*		
A5. Forests and other wooded areas	3.60 (1.09)	0.72	19.69
A6. Natural conservation areas	3.63 (1.04)	0.71	19.29
A7. Outdoor recreation opportunities	3.83 (0.99)	0.78	22.04
A8. Rural landscapes	3.90 (1.02)	0.78	22.05
A9. Opportunities to experience nature	4.01 (0.97)	0.85	25.06
*Agricultural pride* (*α* = 0.91, CR = 0.91, AVE = 0.77)	*3.88 (0.90)*		
A10. Farmland productivity	3.98 (0.99)	0.89	27.84
A11. Fertile soils for growing crops	3.96 (0.99)	0.93	29.61
A12. Agricultural innovation	3.69 (0.97)	0.81	24.03
*Farming lifestyle* (*α* = 0.82, CR = 0.82, AVE = 0.60)	*3.81 (0.82)*		
A13. A sense of responsibility for the land	3.73 (0.99)	0.82	24.02
A14. Freedom to work independently	3.83 (0.94)	0.77	21.82
A15. Ability to work hard to make a living where you live	3.85 (0.92)	0.72	19.66
**Place attachment** ^**2**^	**3.62 (0.91)**		
*Place identity* (*α*^1^ = 0.89, CR^1^ = 0.89, AVE^1^ = 0.73)	*3.74 (0.98)*	*0.97*	*5.80*
B1. The area where I live means a lot to me	3.88 (1.04)	0.84	6.16
B2. I am very attached to the area where I live	3.71 (1.12)	0.86	6.13
B3. I identify strongly with the area where I live	3.64 (1.09)	0.85	6.20
*Place dependence* (*α* = 0.93, CR = 0.93, AVE = 0.83)	*3.32 (1.11)*	*0.87*	*14.72*
B4. I enjoy living here more than any other area	3.41 (1.16)	0.92	19.54
B5. I get more satisfaction out of living here than living in any other place	3.33 (1.19)	0.93	19.57
B6. Living here is more important than living in any other Place	3.20 (1.19)	0.88	18.97
*Social bonding* (*α* = 0.85, CR = 0.76, AVE = 0.59)	*3.81 (0.96)*	*0.90*	*8.72*
B7. I have a lot of fond memories with other people in the area where I live	3.90 (1.09)	0.71	9.04
B8. I have a special connection to the area where I live and the people who live here	3.78 (1.09)	0.80	9.69
B9. I bring my family and friends to the area where I live	3.75 (1.07)	0.78	9.76
**Anticipated emotions** ^**3**^			
*Pride* (*α* = 0.93, CR = 0.93, AVE = 0.73)	*3.63 (0.99)*		
D1. Proud	3.65 (1.09)	0.86	26.31
D2. Accomplished	3.64 (1.09)	0.90	28.42
D3. Satisfied	3.70 (1.12)	0.90	28.36
D4. Worthwhile	3.65 (1.14)	0.83	24.59
D5. Confident	3.53 (1.14)	0.79	23.00
*Guilt* (*α* = 0.97, CR = 0.97, AVE = 0.88)	*3.47 (1.34)*		
E1. Guilty	3.49 (1.41)	0.95	31.44
E2. Remorseful	3.45 (1.39)	0.95	31.38
E3. Sorry	3.53 (1.39)	0.94	31.23
E4. Ashamed	3.42 (1.46)	0.93	30.03
E5. Bad	3.43 (1.44)	0.92	29.90
**Pro-environmental behavior** ^**4**^	**3.53 (0.83)**		
*Conservation lifestyle* (*α* = 0.84, CR = 0.84, AVE = 0.64)	*3.94 (0.89)*	*0.76*	*12.11*
F1. Recycle paper, plastic, and metal in the area where I live	4.07 (1.07)	0.74	15.53
F2. Conserve water or energy in the area where I live	3.96 (0.97)	0.82	16.71
F3. Buy environmentally friends and/or energy efficient Products	3.79 (1.02)	0.84	18.68
*Social environmentalism* (*α* = 0.88, CR = 0.88, AVE = 0.72)	*3.16 (1.06)*	*0.84*	*11.98*
F4. Work with others in the area where I live to address an environmental problem or issue	3.25 (1.12)	0.82	15.77
F5. Participate as an active member in an environmental group in the area where I live	3.03 (1.19)	0.87	15.62
F6. Talk to others in the area where I live about an environmental problem	3.20 (1.19)	0.85	16.14
*Environmental citizenship* (*α* = 0.79, CR = 0.80, AVE = 0.57)	*3.48 (0.96)*	*0.97*	*2.71*
F7. Signed a petition about an environmental issue in the area where I live	3.57 (1.16)	0.81	2.82
F8. Vote to support a policy or regulation that supports environmental protection in the area where I live	3.75 (1.12)	0.79	2.81
F9. Donate money to support environmental protection in the area where I live	3.12 (1.16)	0.66	2.84

Fit for measurement model: *χ*^2^ = 1710.91, *p* < 0.001, df = 824, CFI = 0.96, RMSEA = 0.04, SRMR = 0.04.

1 Statistical symbols: α = Cronbach’s alpha; CR = Composite reliability; AVE = Average variance extracted; λ = Factor loading.

2 Items rated on scale from 1 = Strongly disagree to 5 = Strongly agree.

3 Items rated on scale from 1 = Not at all to 5 = Very much.

4 Items rated on scale from 1 = Very unlikely to 5 = Very likely.

### 4.3. Data analysis

We evaluated possible changes to the original factor structure of the place meaning scale developed by Evans et al. (2022), by conducting an exploratory factor analysis (EFA). First, we found that the survey items had an acceptable Kaiser-Meyer-Olkin Measure of Sampling Adequacy (Kaiser, 1974) value of 0.96 and Bartlett’s test of Sphericity (Bartlett, 1950) was statistically significant (*χ*^2^ = 13,298, *p* < 0.001), which indicated the survey items were appropriate for factor analysis. We then conducted a parallel analysis (Hayton et al., 2004) to identify the number of factors that should be extracted. This analysis suggested the presence of four factors in the place meaning survey items. A final EFA using a four-factor solution with an oblimin rotation (Costello and Osborne, 2005) produced factors, each of which had an eigenvalue above one. These resulting factors were utilized in subsequent confirmatory factor analysis (CFA) alongside the other survey scales.

We tested a series of hypothesized relationships using a two-step modeling approach (Anderson and Gerbing, 1988) to understand the relationships among latent variables included in our structural equation model (Table 1 within the Supplementary material). First, we used CFA to assess model fit and evaluate the psychometric properties of our survey scales. After establishing a measurement model, we then tested our hypotheses in a structural model. Fit of the model to our sample data was evaluated using a chi-square test (Kline, 2015), comparative fit index (CFI > 0.90; Hu and Bentler, 1999), root mean square error of approximation (RMSEA ≤ 0.08; MacCallum et al., 1996), and standardized root mean square residual (SRMR ≤ 0.08; Hu and Bentler, 1999). All constructs had adequate internal consistency (Cronbach’s alpha >0.70; Nunnaly and Bernstein, 1994), average variance extracted (AVE > 0.50; Fornell and Larcker, 1981), and composite reliability (CR > 0.60; Bagozzi and Yi, 1988). After verifying fit for our structural model, we estimated a mediation model to test the indirect effects among constructs. This was performed by bootstrapping indirect effects from 1,000 bootstrap samples, to calculate 95% bias corrected confidence intervals for each effect (Cheung and Lau, 2008; Williams and MacKinnon, 2008). We conducted all analyses in R (version 3.6.1), and performed structural modeling using the ‘lavaan’ package (version 0.6-5; Rosseel, 2012).

## 5. Results

### 5.1. Survey respondent characteristics

Our survey respondents were mostly White (82.9%) and female (65.3%). The average age was 41 years (*SD* = 15.6; see Table 3). Nearly three quarters of respondents had some college education (70.8%) and half had an income less than $50,000. Respondents indicated they had lived in the state of Illinois for an average of 33 years (*SD* = 18.5) and in their current residence for 10 years (*SD* = 10.5). In comparison with the U.S. Census, this survey over-represents non-White residents of the watershed (USAFacts, 2022) but it should be emphasized that the watershed area is predominantly White in its composition.

**Table 3:** Socio-demographic characteristics of respondents (*n* = 614).

Variable	Percent (%)
Gender	
Female	65.3
Male	34.7
Race	
American Indian	2.3
Asian	2.4
White	82.9
Black or African American	12.4
Hispanic	3.7
Other	1.8
Prefer not to answer	1.1
Educational attainment	
No degree	2.0
High school graduate or GED	27.2
Some college	38.6
Bachelor’s degree	18.8
Post-graduate degree	13.4
Annual Income	
Less than $24,999	25.1
$25,000–$49,999	25.1
$50,000–$74,999	15.7
$75,000–$99,999	11.8
$100,000–$124,999	6.4
$125,000–$149,999	3.9
$150,000–$174,999	1.8
$175,000–$199,999	0.8
Over $200,000	1.8
Prefer not to answer	7.5
Age (*M, SD*)	41.4 (15.6)
Years living in Illinois	32.6 (18.5)
Years living in current residence	9.7 (10.5)

Results showed that respondents had strong connections to places in the Kaskaskia River Watershed. Specifically, broad agreement with all survey items measuring place attachment was reported (*M* = 3.62, *SD* = 0.91). The meanings of places evaluated in this study also resonated with survey respondents, as they agreed with statements that comprised the dimensions of natural environment (*M* = 3.79, *SD* = 0.84), agricultural pride (*M* = 3.88, *SD* = 0.90), farming lifestyle (*M* = 3.81, *SD* = 0.82), and family and community legacy (*M* = 3.73, *SD* = 0.87). Our assessment of anticipated emotions showed agreement with survey items that measured pride (*M* = 3.63, *SD* = 0.99) and guilt (*M* = 3.47, *SD* = 1.34). Finally, performance of PEB was high and variable. Respondents were most likely to intend to adopt conservation lifestyles (*M* = 3.94, *SD* = 0.89) and environmental citizenship (*M* = 3.48, *SD* = 0.96), while reporting lowest intentions to engage in social environmentalism (*M* = 3.16, *SD* = 1.06).

### 5.2. Measurement and structural model

Our two-step analysis indicated adequate model fit for the measurement model (*χ*^2^ = 1866.88, *p* < 0.001, df = 837, CFI = 0.95, RMSEA = 0.04, SRMR = 0.04) and structural model (*χ*^2^ = 2287.72, *p* < 0.001, df = 1,099, CFI = 0.95, RMSEA = 0.05, SRMR = 0.08). In our measurement model, all factor loadings had acceptable values (λ > 0.40; Hair et al., 1998) and all latent constructs had acceptable measures of internal consistency and validity. All hypotheses in the structural model were supported except for the relationship between anticipated guilt and PEB (Table 4). Notably, we observed significant direct correlations of all place meanings with place attachment including the effects of family and community legacy (γ = 0.47, *p* < 0.001), natural environment (γ = 0.26, *p* < 0.001), agricultural pride (γ = −0.14, *p* = 0.027), and farming lifestyle (γ = 0.29, *p* = 0.022) meanings. We observed that place attachment had a direct positive correlation with PEB (*β* = 0.13, *p* = 0.003). Another finding was that place attachment had a significant positive correlation with both anticipated pride (*β* = 0.36, *p* < 0.001) and guilt (*β* = 0.20, *p* < 0.001). In turn, anticipated pride had a significant positive correlation with PEB (*β* = 0.53, *p* < 0.001), which was stronger than the effect of anticipated guilt on PEB (*β* = 0.12, *p* = 0.001). Overall, the hypothesized model accounted for 70% of the variance in place attachment and 37% of the variance in PEB. Finally, mediation analyses indicated that place attachment had a significant and positive indirect effect on PEB through anticipated pride {*β* = 0.19 (95% CI [0.14, 0.25]), *p* < 0.001}. In contrast, the indirect effect of place attachment on PEB through anticipated guilt was not statistically significant {*β* = 0.02 (95% CI [0.001, 0.05]), *p* = 0.053}.

**Table 4:** Results from the latent variable model.

Dependent variables	Predictor variables	*Std. Coef.*	SE	*z*-value	*R* ^2^
Place attachment	Family and community legacy meanings	0.47***	0.07	6.24	0.70
	Natural environment meanings	0.26***	0.09	3.54	
	Agriculture pride meanings	−0.14*	0.06	−2.21	
	Farming lifestyle meanings	0.29*	0.16	2.28	
Anticipated pride	Place attachment	0.36***	0.05	8.38	0.13
Anticipated guilt	Place attachment	0.20***	0.07	4.74	0.04
Pro-environmental behavior	Place attachment	0.13**	0.03	3.17	0.37
	Anticipated pride	0.53***	0.03	8.92	
	Anticipated guilt	0.12**	0.02	1.45	

Fit for structural model: *χ*^2^ = 1866.88, *p* < 0.001, df = 1,099, CFI = 0.95, RMSEA = 0.05, SRMR = 0.08.

* *p* < 0.05;

** *p* < 0.01;

*** *p* < 0.001.

## 6. Discussion

This study explored the effects of multiple predictors of PEB, particularly concepts of place and discrete emotions, among residents of an agricultural watershed in the Midwestern USA. Our hypothesized model was largely supported, and we effectively distinguished between place meanings and place attachment. Four dimensions of place meanings – including farming lifestyles, agricultural pride, natural environments, and family and community legacy – emerged as explanatory variables that correlated with attachment. Extending previous research (Stedman, 2003; Larson et al., 2018; Rajala et al., 2020), we suggest that place meanings are situated as an antecedent to place attachment, which in turn, explains other psychological phenomena including PEB. One key finding was that *all* place meanings were positively correlated with attachment. This observation lies in contrast to previous research where place-based concepts have been deeply contested. For example, Stedman (2003) studied a rural lake-dominated landscape and found that “community” and “wilderness” based meanings negatively predicted place attachment. Whereas social conflicts were more prevalent in this study given the transition from nature-based to more community-based meanings, our study context did not indicate that human-place bonding was underpinned by feelings of unrest, perhaps because traditional rural agricultural meanings held sway.

We observed direct effects of place attachment on PEB, which aligns with a substantive body of previous work (Vaske and Kobrin, 2001; Halpenny, 2010; Scannell and Gifford, 2010; Daryanto and Song, 2021). These findings indicate that place attachment corresponds to the promotion of behaviors that mitigate threats to landscape change (Devine-Wright, 2013). We also observed indirect effects between two place meanings and PEB, in that meanings tied to the notion of legacy and the natural environment offered explanations for intended behavior (Stedman, 2016; Larson et al., 2018). In other words, it is important to first understand how people define their relationships with an environment before gauging the strength of connections formed between people and places. Thus, both concepts of place examined in this study were instrumental in understanding preferences for and reactions to social and ecological change (Oteros-Rozas et al., 2015; Masterson et al., 2017; Kendal and Raymond, 2019).

We found partial support for a series of hypotheses about emotional concepts mediating the relationship between place attachment and PEB. Our findings indicated that anticipated emotions were distinguishable from place attachment, which brings clarity to previous research suggesting that place attachment, particularly the affective dimension of attachment, is an emotional concept (Low and Altman, 1992; Jorgensen and Stedman, 2001; Kyle et al., 2004; Ramkissoon et al., 2013). More specifically, we found that both pride and guilt predicted PEB but that pride had greater predictive power (Onwezen et al., 2013; Han et al., 2017; Shipley and van Riper, 2022). This finding extends previous research that has argued feelings of anticipated pride are more strongly associated with place-based PEB than anticipated guilt, which has important implications for research that seeks to leverage the connections between people and places to inspire environmental stewardship and strengthen the health benefits of nature (Browning et al., 2019).

We observed that place attachment predicted both pride and guilt, suggesting that the strength of a connection between people and places contributes to experiences that are drawn upon when anticipating these two discrete emotions. Interestingly, anticipated pride partially mediated the relationship between attachment and PEB while anticipated guilt did not. This result provides insights into the possible mechanisms underpinning the attachment-PEB relationship (Halpenny, 2010). Given that people generally strive to feel positive rather than negative emotions, it is possible that pride mediated the relationship while guilt did not because people were more likely to associate feelings of pride with the places to which they were attached. Respondents were also more likely to intend to engage in behaviors that protected rather than harmed places, which sheds further light on why pride may have mediated the attachment-PEB relationship. Overall, this study demonstrated that concepts of place function as a basis for emotions that a person anticipates experiencing when making behavioral decisions.

## 7. Conclusions and implications

We report on findings from a model that accounts for the combined effects of place meanings on attachment, which in turn, influences two emotional concepts that predict behavioral intentions. To date, the extant literature has largely supported the idea that “affect” is a dimension of place attachment (Jorgensen and Stedman, 2001; Williams and Vaske, 2003; Kyle et al., 2004). We extend this argument by providing empirical evidence of the differences between place attachment and discrete emotions – a distinction that will improve the predictive capacity of models focused on explaining patterns of PEB. Distinguishing between these constructs is novel, because of the differential effects of pride and guilt on PEB. We further argue that the anticipated emotion of pride warrants greater research attention because it mediates the relationship between attachment and PEB, which lies in contrast to previous research that has emphasized the primary role of guilt as a motivator for behavior change (Elgaaied, 2012). We thus bring conceptual clarity to a set of interrelated psychological phenomena to advance knowledge of how human-place bonding can facilitate or constrain actions intended to benefit the environment.

This article shows that there are positive relationships among place-related predictors of PEB to support more effective environmental policy initiatives that respond to the ways that people think, feel, and act in response to landscape change. Concepts of place are particularly important to consider in the decision-making process because they serve as a root cause of place-protective behaviors (Devine-Wright, 2009; Rajala et al., 2020). Creating and maintaining opportunities to strengthen human-place bonds and alleviate concerns about degradation will amplify the effectiveness of environmental outreach campaigns targeted at building responsiveness within a constituency. On one hand, tourists and visitors can be encouraged to participate in PEBs through messaging that activates feelings of pride and guilt, which will work in concert with place attachment that positively correlates with behavioral outcomes (Halpenny, 2010). On the other hand, residents can also be encouraged to act in ways that benefit the environment, given that PEBs performed by this stakeholder group tend to be relatively lower (Daryanto and Song, 2021; Andrade et al., 2022). Environmental communication strategies should therefore consider multiple pathways for encouraging environmental protection among a range of interest groups (Golebie and van Riper, 2022), particularly through the power of emotions as a mechanism for behavior change. Building pride in local places shows particular promise as a way to promote environmental stewardship and foster place attachment over time.

This study provides both theoretical and managerially relevant implications for future research. Theoretically, we demonstrated that place meanings, place attachment, pride, and guilt are all distinguishable but interrelated phenomena using latent variable modeling techniques. We also deepen understanding of the ways in which place attachment shapes PEB, primarily through the mediating effects of pride and guilt. The empirical evidence we bring to bear clarifies how affect can function as a place-related concept when it is operationalized and connected to the science of emotions. A key managerial implication of this study is the utility of discrete emotions for promoting connections to landscapes among diverse stakeholders. Negative emotions have traditionally been positioned as key mechanisms for motivating people to protect the environment (e.g., feeling guilt about contributing to climate change, Elgaaied, 2012); while no doubt a powerful catalyst, research also suggests that repeated experiences of negative emotions can have diminishing effects on behavior (O'Neill and Nicholson-Cole, 2009). We therefore underscore the arguments made in more recent studies that have suggested positive emotions such as pride are more effective for promoting continued and sustained engagement in PEB (Venhoeven et al., 2020). By elucidating which emotions are most strongly associated with PEB, and that mediate the place attachment-PEB relationship, programs and interventions focused on behavior change can be better developed.

## Data availability statement

The datasets presented in this study can be found in online repositories. The names of the repository/repositories and accession number (s) can be found at: http://dx.doi.org/10.5281/zenodo.5851726.

## Ethics statement

The studies involving human participants were reviewed and approved by University of Illinois Institutional Review Board. The patients/participants provided their written informed consent to participate in this study.

## Author contributions

NS conceptualized idea, data collection and analysis, and developed first draft. CVR conceptualized idea, provided funding, project administration, offered conceptual guidance, and reviewed and edited manuscript. WS provided funding, project administration, offered conceptual guidance, and reviewed and edited manuscript. MC provided funding, offered conceptual guidance, and reviewed and edited manuscript. RS and FD offered conceptual guidance and reviewed and edited manuscript. All authors contributed to the article and approved the submitted version.

## Funding

This research was conducted under the support of United States Department of Agriculture National Institute of Food and Agriculture grant (#2018–68002-27918) and University of Illinois, College of Agricultural, Consumer, and Economic Sciences Future Interdisciplinary Research Explorations (grant #ILLU-741-380).

## Conflict of interest

The authors declare that the research was conducted in the absence of any commercial or financial relationships that could be construed as a potential conflict of interest.

## Publisher’s note

All claims expressed in this article are solely those of the authors and do not necessarily represent those of their affiliated organizations, or those of the publisher, the editors and the reviewers. Any product that may be evaluated in this article, or claim that may be made by its manufacturer, is not guaranteed or endorsed by the publisher.
