# Supplementary material for: Pride and guilt as place-based affective antecedents to pro-environmental behavior
Source: Front Psychol. 2023 Jan 19;13:1084741. doi: 10.3389/fpsyg.2022.1084741 (PMC9892858; doi:10.3389/fpsyg.2022.1084741)
Supplement: Supplementary file 1 [file Table_1.DOCX]

Supplementary Material

| **Table 1.** Estimated correlation matrix among latent factors included in the structural equation model | | | | | | | | |
| --- | --- | --- | --- | --- | --- | --- | --- | --- |
|  | 1 | 2 | 3 | 4 | 5 | 6 | 7 | 8 |
| 1. Family & community legacy | - |  |  |  |  |  |  |  |
| 1. Natural environment | .702*** | - |  |  |  |  |  |  |
| 1. Agricultural pride | .647*** | .762*** | - |  |  |  |  |  |
| 1. Farming lifestyle | .835*** | .839*** | .816*** | - |  |  |  |  |
| 1. Place attachment | .792*** | .712*** | .586*** | .773*** | - |  |  |  |
| 1. Anticipated pride | .340*** | .328*** | .270*** | .355*** | .336*** | - |  |  |
| 1. Anticipated guilt | .140** | .220*** | .198*** | .191*** | .178*** | .474*** | - |  |
| 1. Pro-environmental behavior | .357*** | .367*** | .295*** | .365*** | .298*** | .612*** | .369*** | - |

** *p* < .01, *** *p* < .001
